# Bilateral adrenal haemorrhages: a crucial incidental finding

**DOI:** 10.1259/bjrcr.20160107

**Published:** 2016-12-08

**Authors:** Soo-Min Cho, Peter Bye

**Affiliations:** ^1^Department of PET and Nuclear Medicine, Royal Prince Alfred Hospital, Sydney, NSW, Australia; ^2^Department of Respiratory Medicine, Royal Prince Alfred Hospital, Sydney, NSW, Australia; ^3^Sydney Medical School, University of Sydney, Sydney, NSW, Australia

## Abstract

Adrenal haemorrhage is a rare condition that has the potential to cause life-threatening adrenal insufficiency, especially if it affects both the adrenal glands. The difficulty in diagnosing adrenal haemorrhages lies in the non-specific clinical presentation including hypotension and abdominal pain. The following case report demonstrates the possible clinical presentations of non-traumatic adrenal haemorrhages and the method of diagnosing and treating adrenal insufficiency. In a medical era where overdiagnosis and “incidentalomas” are becoming more prevalent, this case nicely demonstrates the fortunate use of imaging to detect a potentially life-threatening condition.

## Case presentation

A 50-year-old male presented to the emergency department with sudden onset right upper quadrant (RUQ) abdominal pain. It was described as a colicky sharp stabbing pain, which progressed to a constant dull discomfort, worse with coughing and deep inspiration. This was preceded by a 1 week history of productive cough with yellow sputum. His past medical history included absence of trauma, undifferentiated immunodeficiency, idiopathic thrombocytopaenia (ITP) with splenectomy, a previous possible stroke, and paroxysmal atrial fibrillation on warfarin. He had multiple courses of antibiotics in the past for recurrent lower limb cellulitis and respiratory tract infections post splenectomy.

The patient weighed 152 kg, with a body mass index of 45. His vital signs were stable. There was pain in the RUQ on palpation. Blood test revealed a platelet count of 232  × 10^9^^ ^l^−1^, mildly elevated white cell count of 13.8 × 10^9^ l^−1^ and C-reactive protein of 12.7 mg l^−1^. Liver function tests were normal. International normalized ratio (INR) was elevated at 3.8. Lactate was elevated at 2.7 mmol l^−1^. Possible differentials included acute cholecystitis and right lower lobe pneumonia with associated pleurisy.

**Figure 1.**
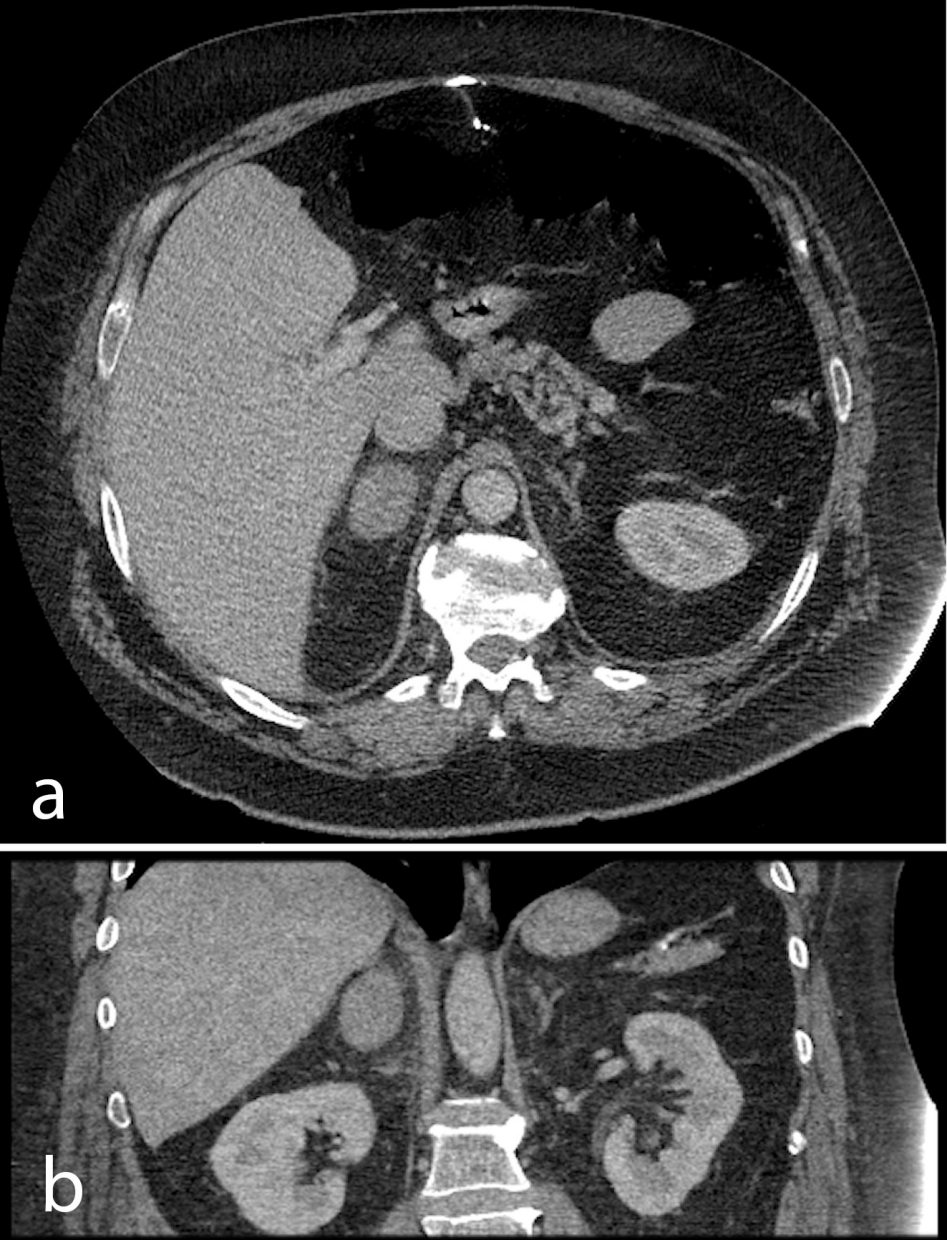
Initial CT scan: portal venous phase images. (a) Axial and (b) coronal CT images showing an enlarged right adrenal gland and a normal “Y”-shaped left adrenal gland.

The patient’s body habitus was deemed unsuitable for a reliable abdominal ultrasound; therefore, the patient underwent a CT abdomen and pelvis with oral and intravenous contrast. Portal venous phase and 10 min delayed phase were obtained. The study revealed a well-defined ovoid mass of 37 × 27 mm with Hounsfield Unit (HU) of 51 (both in portal venous phase and delayed phase) with no washout, suggestive of an adrenal lesion (Figure 1). The left adrenal was of normal “Y” shape in appearance. There was no evidence of cholecystitis. There was patchy consolidation in the right lower lobe. The patient was commenced on intravenous antibiotics for a lower respiratory tract infection.

**Figure 2.**
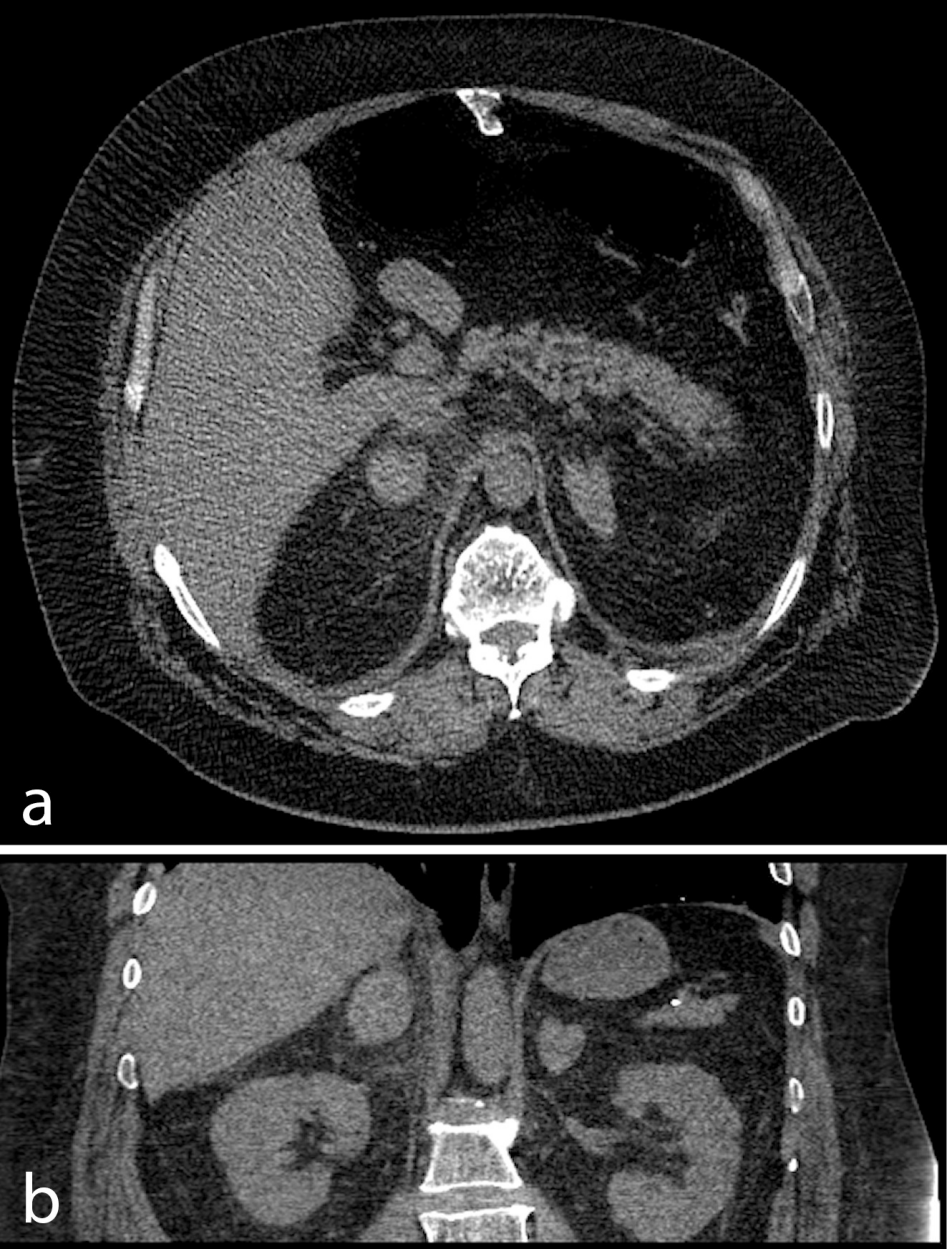
Repeat non-contrast CT scan. (a) Axial and (b) coronal CT images showing bilateral adrenal gland enlargements.

Over 12 h the pain had migrated from the RUQ to the left flank. The patient underwent a further non- contrast CT scan of abdomen and pelvis with portal venous phase (Figure 2). The study showed persistent right adrenal mass of 40 × 31 mm with a HU of 36. There was a new enlargement in the left adrenal gland measuring 34 × 24 mm with a HU of 25. The sudden enlargement of the left adrenal gland strongly suggested acute haemorrhage. To further evaluate this adrenal finding, an adrenal CT protocol was carried out with pre, arterial, portal venous and 10 min delayed images, 1 week after the initial scan. There was no discrete adrenal mass and densities of adrenals were consistent in all phases, measuring 40  HU.

## Treatment

The effect of warfarin was reversed with vitamin K and fresh frozen plasma, bringing down the INR to 1.3. The patient became hypotensive and a Synacthen test confirmed primary adrenal insufficiency. He was immediately commenced on intravenous hydrocortisone and fludrocortisone. Given the adrenal haemorrhages, the need for warfarin was re-evaluated. Previously, the patient presented with vertigo and atrial fibrillation and was investigated for stroke. The CT scan of brain was normal. Owing to his obesity, he was unable to undergo an MRI examination. Given that he was in atrial fibrillation at the time of presentation, he was commenced on warfarin.

**Figure 3.**
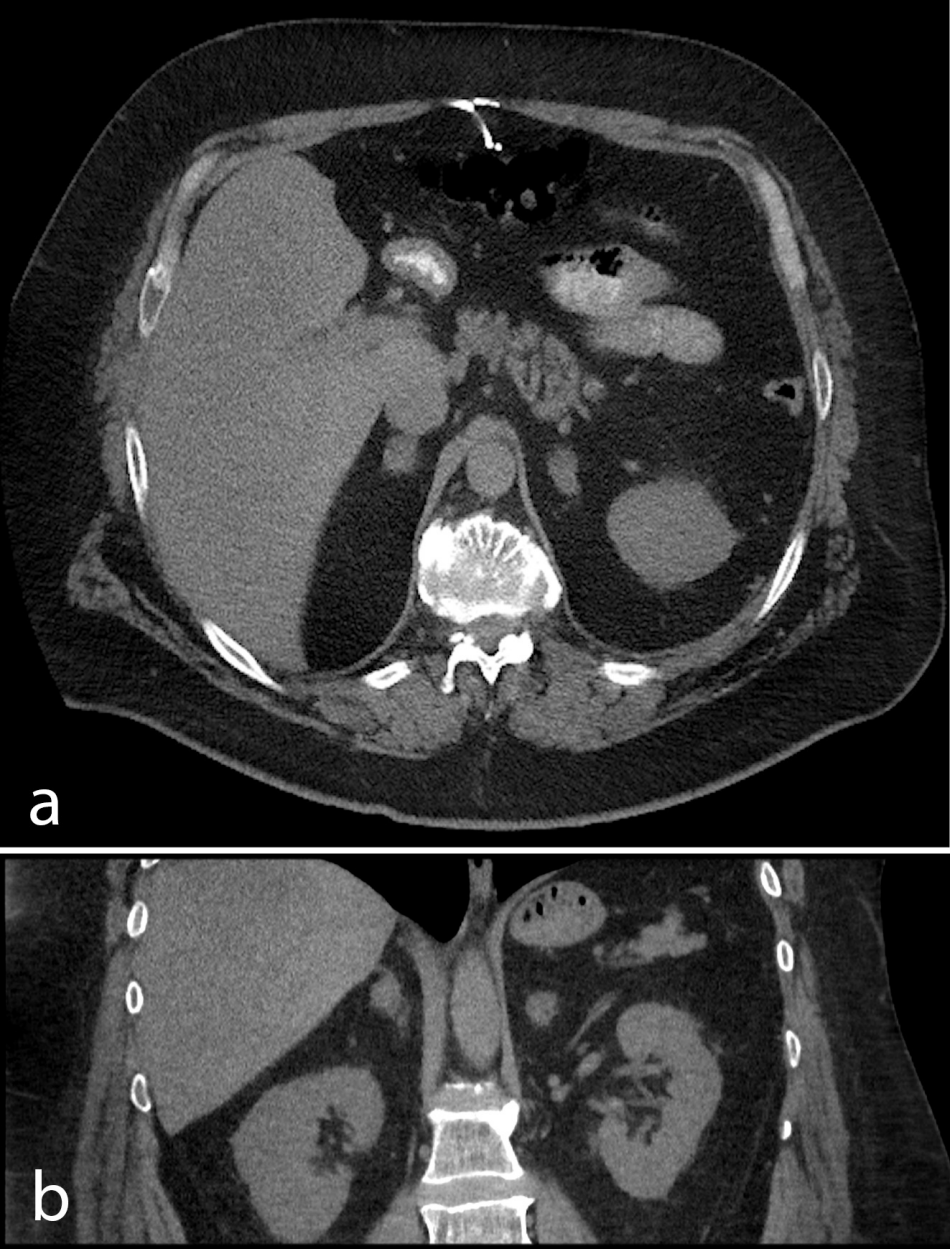
Follow-up CT scan: non-contrast images. (a) Axial and (b) coronal CT images showing a reduction in size of bilateral adrenal gland enlargements.

On this presentation, an MRI scan of brain was performed as he had lost weight. The MRI scan did not reveal any evidence of stroke. The decision was made not to recommence the patient on warfarin. The patient recovered from his lower respiratory tract illness and was discharged on oral hydrocortisone and fludrocortisone. A follow-up pre, portal venous and delayed phase CT scan 2 months later revealed a reduction in size of adrenals as well as resolution of right lobe consolidation and the lung nodule (Figure 3).

## Discussion

Adrenal haemorrhage is a rare condition with the potential to cause life-threatening adrenal insufficiency, especially if it affects both the adrenal glands. The difficulty in diagnosing adrenal haemorrhages lies in the non-specific clinical presentation including hypotension and abdominal pain. Prior to advances in imaging the majority of the diagnoses were made during post-mortem studies.^1^ Non-traumatic adrenal haemorrhage is observed in times of physiological stress, such as recent surgery, hypotension, sepsis (*e.g*. fulminant meningococcaemia in Waterhouse–Friderichsen syndrome) and burns.^1–5^

Multiple predisposing factors have been linked to non- traumatic adrenal haemorrhage including sepsis, hypotension, recent surgery, anticoagulation and antiphospholipid syndrome.^6^ This patient had a history of idiopathic thrombocytopaenia; however, his platelets were within normal range and despite a mildly elevated INR from warfarin therapy, did not have any history of bleeding. Waterhouse–Friderichsen syndrome is another dramatic cause of adrenal haemorrhage, where meningococcal sepsis leads to bleeding in the adrenals and subsequent failure.^3,7^ Although this patient had a concurrent lower respiratory tract infection, he remained afebrile with no clinical signs of sepsis along with negative blood cultures. The recurrent nature of his lower limb cellulitis is another potential source of chronic infection.

An abdominal ultrasound would be the first choice in investigating an RUQ pain in an outpatient setting. Although adrenals can be well identified in infants and children using an ultrasound, in adults, often it is difficult to visualize this retroperitoneal structure owing to bowel gas and obesity.^8,9^

There is ongoing research on delineation between benign and malignant adrenal masses on a CT scan, which are mostly performed as part of a staging scan for oncology patients^10^ and found as incidental lesions.^2,8^ It is well described that on a non-contrast CT scan, a diagnosis of a benign adrenal adenoma can be made if the attenuation is less than 10 HU. The rapid rate of CT contrast washout indicates adenomas as opposed to slower washout in malignant masses.^9,10^ The CT scan appearance of adrenal haemorrhages can be variable. Adrenal haemorrhages can appear on a non-contrast CT scan with a high attenuation, with HU of 50–90 during the acute phase. There is a gradual reduction in adrenal size over time and there may be evidence of calcification.^3,11^ In this patient, the acute enlargement of adrenal glands, HU > 10, and a subsequent follow-up scan revealing a reduction in size are most consistent with a diagnosis of haemorrhage.

Although rare, adrenal haemorrhages should be recognized and diagnosed early in patients with risk factors as prompt treatment can be life-saving.

## Learning points

1. With the current widespread use of CT imaging, adrenal haemorrhage is most often identified serendipitously during investigation for other diagnosis.
2. Adrenal haemorrhages are uncommon and are difficult to diagnose as they can present with non-specific clinical symptoms and signs. The diagnosis should be suspected in patients who present with abdominal pain with a history of trauma, recent anticoagulation or evidence of sepsis. Laboratory testing such as Synacthen test can be useful.
3. Appropriate use of CT imaging and various phases can help delineate between benign, malignant or other adrenal lesions. Follow-up imaging is important and further evaluation with biopsy may be warranted if malignancy is suspected.

## Consent

Written informed consent was obtained from the patient for publication of this case report, including accompanying images.
